# A New Method to Quantify Ifosfamide Blood Levels Using Dried Blood Spots and UPLC-MS/MS in Paediatric Patients with Embryonic Solid Tumours

**DOI:** 10.1371/journal.pone.0143421

**Published:** 2015-11-23

**Authors:** Luz-María Torres, Liliana Rivera-Espinosa, Juan L. Chávez-Pacheco, Carlos F. Navas, Joel A. Demetrio, Radamés Alemón-Medina, Francisca Trujillo, Martín Pérez, Martha M. Zapata, Rocío Cárdenas, Citlaltepetl Salinas, Arnoldo Aquino, Rafael Velázquez-Cruz, Manuel-de-Jesús Castillejos

**Affiliations:** 1 Laboratorio de Farmacología, Instituto Nacional de Pediatría, Mexico City, Mexico; 2 Facultad de Estudios Superiores Iztacala, Universidad Nacional Autónoma de México, Mexico City, Mexico; 3 Laboratorio de Neuropatología, Instituto Nacional de Neurología y Neurocirugía, Mexico City, Mexico; 4 Laboratorio de Genómica del Cáncer, Instituto Nacional de Enfermedades Respiratorias, Mexico City, Mexico; 5 Laboratorio de Genómica del Metabolismo Óseo, Instituto Nacional de Medicina Genómica, Mexico City, Mexico; 6 Unidad de Vigilancia Epidemiológica, Instituto Nacional de Enfermedades Respiratorias, Mexico City, Mexico; 7 Servicio de Oncología, Instituto Nacional de Pediatría, Mexico City, Mexico; Johns Hopkins University, UNITED STATES

## Abstract

Ifosfamide blood concentrations are necessary to monitor its therapeutic response, avoiding any adverse effect. We developed and validated an analytical method by UPLC-MS/MS to quantify ifosfamide in dried blood spots (DBS). Blood samples were collected on Whatman 903® filter paper cards. Five 3 mm disks were punched out from each dried blood spot. Acetonitrile and ethyl acetate were used for drug extraction. Chromatographic separation was carried out in an Acquity UPLC equipment with a BEH-C18 column, 2.1 x 100 mm, 1.7 μm (Waters®). The mobile phase consisted in 5 mM ammonium formate and methanol:acetonitrile (40:48:12 v/v/v) at 0.2 mL/min. LC-MS/MS detection was done by ESI+ and multiple reaction mode monitoring, ionic transitions were m/z^1+^ 260.99 > 91.63 for ifosfamide and 261.00 > 139.90 for cyclophosphamide (internal standard). This method was linear within a 100–10000 ng/mL range and it was accurate, precise and selective. Ifosfamide samples in DBS were stable for up to 52 days at -80°C. The procedure was tested in 14 patients, ages 1 month to 17 years (9 males and 5 females), with embryonic tumours treated with ifosfamide, alone or combined, at a public tertiary referral hospital. Ifosfamide blood levels ranged from 11.1 to 39.7 μmol/L at 12 hours after the last infusion, while 24-hour levels ranged from 0.7–19.7 μmol/L. The median at 12 hours was 19.5 μmol/L (Q_25_ 14.4–Q_75_ 29.0) and 3.8 μmol/L (Q_25_ 1.5–Q_75_ 9.9) at 24 hours, p<0.001. This method is feasible to determine ifosfamide plasma levels in paediatric patients.

## Introduction

Cancer is the second most common cause of death [1], and solid tumours account for 60% of all malignancies. Cancer mortality rates increase due to diagnostic delay, tumour aggressiveness, general health condition and the toxicity of the antineoplastic drugs. Ifosfamide is one of the most commonly used drugs in chemotherapy for children and adults with solid tumours [1], as it is a very effective broad-spectrum drug that can be used alone or combined [1]. It has been reported, however, that it can cause severe systemic toxicity or even the death of the patient [1–5]. In the present study, we developed and validated a rapid and sensitive method to measure ifosfamide blood concentrations in paediatric patients, since there is still lacking a method to monitor ifosfamide levels routinely, as it is done with other antineoplastic drugs such as methotrexate, whose toxic effects are also considerable. Knowing that 1 μM methotrexate is achieved in blood at 24 hours [2], allows the prompt implementation of rescue measures in the case this plasma concentration is overtaken, even if toxicity signs are not clinically shown. For these reasons, determination of blood ifosfamide concentrations is essential to provide an individualised treatment, corroborate expected response, and reduce dosage and/or provide rescue measures if necessary, before toxicity is severe or irreversible. Likely, the introduction of blood collection in Whatman 903® filter paper cards (Guthrie cards), allows the use of minimal blood volumes, which represents a great technical advantage in paediatric patients undergoing antineoplastic therapy.

## Material and Methods

### Reagents

Ifosfamide and cyclophosphamide monohydrate pure standards were purchased from Sigma-Aldrich Co® (St. Louis MO, USA) and MP Biomedicals® (Fountain Pkwy, Solon OH, USA), respectively. Standards of etoposide, carboplatin, vincristine sulphate and methotrexate were all purchased from MP Biomedicals® (Fountain Pkwy, Solon OH, USA), and ondansetron from Toronto Research Chemicals Inc® (Toronto, Canada).

LC-MS grade acetonitrile and methanol were obtained from EMD Millipore Co® (Venezuela), HPLC grade ethyl acetate from Merck® (Darmstat, Germany) and propanol from J.T. Baker® (Xalostoc, Mexico). Ammonium formate was purchased from Sigma Adrich® (St. Louis MO, USA). Whatman 903® cotton filter paper (“Guthrie” cards) were bought from GE Healthcare Ltd.® (Cardiff, UK). Milli-Q grade 1 water (Millipore, Molsheim, France) was used to prepare all the solutions and dilutions.

### Chromatographic conditions

An Acquity (Waters Co.®, Milford MA, USA) ultra-performance liquid chromatography (UPLC) equipment coupled with a Micromass Quattro Micro tandem mass spectrometer (Waters Micromass®, Manchester, UK) was used in electrospray positive ionization mode (ESI+) and was controlled by a MassLynx NT 4.0 (Waters Micromass, Beverly, MA, USA) software. Compound separation was achieved using an Acquity UPLC BEH-C18 column, 2.1 x 100 mm, 1.7 μm (Waters Co.®, Milford MA, USA), at 40°C; the autosampler was set at 15°C. Flow rate was 0.20 mL/min. Running time was 3.5 minutes; retention time was 1.9 minutes for ifosfamide and 2.0 minutes for cyclophosphamide. The mobile phase consisted of A) 5 mM ammonium formate and B) methanol (MeOH) and acetonitrile (ACN) 80:20 v/v. Solvent A:B ratio was 40:60 (v/v) in order to obtain a final mobile phase consisting of ammonium formate:MeOH:ACN (40:48:12 v/v/v).

### Spectrometric conditions

Analytes of interest were measured in multiple reaction monitoring mode, ion transitions were m/z^1+^ 260.99 > 91.63 for ifosfamide and 261.00 > 139.90 for cyclophosphamide. Conditions were as follows: Cone Voltage = 25V for both analytes, Collision Energy = 25V for ifosfamide and 20V for cyclophosphamide, with a dwell time of 0.2 seconds. Data obtained were processed with a MassLynx® 4.1 software.

### Standards and controls

Initial ifosfamide and cyclophosphamide 1mg/mL solutions were individually prepared in 100% MeOH. From the initial ifosfamide solution, work solutions were prepared (20X) in 70% MeOH in order to obtain solutions for calibrators at 2, 10, 20, 50, 100, 150 and 200 μg/mL concentrations. Solutions for low, medium, and high quality control points were prepared at 6, 80, and 160 μg/mL respectively. Independent calibration curves (100, 500, 1000, 2500, 5000, 7500 and 10000 ng/mL) were prepared by adding 475 μL of total blood (haematocrit 30% and 45%) and 25 μL of ifosfamide solution at the different concentrations (20X). 40 μL of the mix were poured onto the Whatman 903 filter paper cards in each circle, separately. Cards were allowed to dry at room temperature for 6 hours, then were labelled and stored in low gas-permeability plastic bags including desiccant packs at 4°C and -80°C until analysed. Quality control points at low, medium and high levels (300, 4000 and 8000 ng/mL, respectively) were prepared with the same procedure as the calibration curves, using the same initial solutions (20X).

### Sample processing (extraction)

Five 3 mm diameter disks were punched out of every blood spot on the card with a manual McGill, Inc. puncher. Disks were placed into 1.5 mL microtubes where 20 μL of cyclophosphamide was added (300 ng/ml in 70% MeOH) and then shaken in a vortex mixer for 10 seconds. 100 μL ACN and 200 μL ethyl acetate were added to the tubes and underwent vortex mixing at maximum speed for two minutes as well as sonicating for five minutes at room temperature. Dried blood samples were centrifuged for five minutes at 12000 rpm at room temperature. The organic phase was decanted into a different tube and it was evaporated at 40°C under airflow. 200 μL of a water:MeOH:ACN mixture (60:32:8, v/v/v) were added to the sample and 8 μL of this resuspended sample were injected into the chromatographic system.

### Method application

Fourteen paediatric patients attended in a public tertiary referral hospital, ages from 1 month to 17 years were enrolled in this study. Parents and responsible relatives signed an informed consent. Children over 12 signed an informed assent form. All of the patients had been diagnosed with solid embryonic tumours and were undergoing chemotherapy with ifosfamide, alone or combined with carboplatin, etoposide, vincristine or methotrexate. The patients received 1.8 g/m^2^/day ifosfamide for 2, 3 or 5 days along with mesna 1.8 g/day and hydration with saline solution (0.9%). The present study was approved by the research and ethics committees of our institute (INP, National Paediatrics Institute), with registry number 068/2013.

Solid embryonic tumours included: retinoblastoma, germ cell tumour, osteosarcoma, Ewing sarcoma, hepatoblastoma and astrocytoma.

Blood samples were taken 12 and 24 hours after ifosfamide infusion on the last day of chemotherapy, by finger puncture with BD Microtainer® lancets, in Whatman 903 filter paper cards. These cards were allowed to dry in the dark, protected from sunlight and at room temperature, for six hours in horizontal position, then were placed into low gas permeability plastic bags (Ziploc®) along with desiccant packs, and were stored at -80°C until analysed.

### Statistical analysis

Mean ± standard deviation and the coefficient of variation were determined to assess the precision of our procedure. Ifosfamide concentration medians at 12 and 24 hours were compared using the Wilcoxon’s test. Statistically significant differences were those with a p < 0.005.

## Results

### Method development

During the optimization of chromatographic conditions, several mobile phases and solution ratios, such as 0.1% and 1% formic acid in water, 2 mM ammonium acetate, and 5 mM ammonium formate mixed with ACN and MeOH, were tested individually or in different proportions. In the course of optimizing ionization (tuning), the best was achieved with 5 mM ammonium formate combined with MeOH in a 40:60 v/v ratio. For compound extraction, direct precipitation with MeOH and ACN and liquid-to-liquid extraction with ethyl acetate:chloroform 80:20 v/v (as the only extraction agent) and ethyl acetate were tested. The cleanest and most efficient extraction was achieved with ethyl acetate. However, to improve the purity of the sample, we added ACN, so the best extraction was obtained with both ACN and ethyl acetate (33.3:66.6 or 1:2 ratio v/v).

### Haematocrit effect

The effect of red blood cell density was determined by quantifying the ifosfamide concentration in blood samples with different percentages of haematocrit (20, 25, 30, 35, 40, 45 and 50%). All the samples contained the same ifosfamide concentration (5000 ng/mL). DBS samples were quantified using two calibration curves; one curve was constructed with 30% haematocrit and the other with 45% haematocrit. After quantifying the samples with the 30% haematocrit curve, samples with haematocrit from 20 to 35% showed percentages of deviation lower than 15% (Table 1), whereas samples with haematocrit from 40 to 50% showed percentages of deviation of 24.98, 28.93 and 35.1%. Additionally, samples with haematocrits between 35 and 50% can be quantified with a calibration curve at a 45% haematocrit curve, with a deviation lower than 10%. This suggests that drug concentrations contained in the samples may be underestimated if their haematocrit percentages are not in accordance with the curve. Since the reported haematocrit in paediatric patients ranges from 30 to 45%, we decided to validate this method using these values.

**Table 1:** Influence of the haematocrit on ifosfamide blood determination.

	Sample haematocrit (%)						
A. 30% HTC	20	25	30	35	40	45	50
Mean ± SD (ng/mL)	4528.9 ± 54.0	4830.1 ± 38.3	4993.2 ± 18.3	5162.6 ± 121.8	6249.2 ± 266.8	6446.7 ± 340.3	6755.0 ± 460.4
% Deviation	-9.4	-3.39	-0.14	3.25	24.98	28.93	35.1
A. 45% HTC							
Mean ± SD (ng/mL)	3690.0 ± 156.4	3861.6 ± 139.5	4315.2 ± 151.7	4829.8 ± 77.7	4899.3 ± 59.4	4993.0 ± 69.1	5119.8 ± 116.5
% Deviation	-26.20	-22.76	-13.69	-3.40	-2.01	-0.13	2.39

SD: Standard Deviation, % Deviation after triplicate quantification of a nominal concentration (5000 ng/mL) with a calibration curve constructed with 30% (A) and 45% (B) haematocrit, it was calculated as: (theoretical concentration minus calculated concentration/theoretical concentration)*100 [6].

### Method validation

Validation parameters were determined according to the Mexican Official Guidelines (NOM-177-SSA1-2013 [6], which are in accordance with the international guidelines for bioanalytical methods [7, 8].

#### Linearity

The method was linear over the range of 100–10,000 ng/mL, and was evaluated by constructing calibration curves (n = 3/day) on three consecutive days (Table 2).

**Table 2:** Linearity of the UPLC-MS/MS method for ifosfamide quantification in DBS with 30 and 45% HTC.

**N**	**Ifosfamide concentration (ng/mL) with 30% HTC**						
	**LLQ (100)**	**500**	**1000**	**2500**	**5000**	**7500**	**10000**
**1**	94.9	521.3	972.1	2723.9	4588.06	7500.2	10190.1
**2**	100.4	500.8	997.5	2594.5	4722.2	7397.3	10293.1
**3**	95.5	545.6	1008.4	2461.8	4553.8	7605.3	10312.3
**Mean ± SD**	96.9±3.0	522.5±22.4	992.6±18.6	2593.4±131.0	4621.3±89.0	7500.9±104.0	10265.1±65.7
**% CV**	3.1	4.2	1.8	5.0	1.9	1.3	0.6
**% Deviation**	3.0	-4.5	0.7	-3.7	7.5	-0.01	-2.6
	**Ifosfamide concentration (ng/mL) with 45% HTC**						
**1**	97.6	517.0	1042.1	2325.1	5247.9	7231.9	10139.3
**2**	94.5	494.8	1066.0	2483.2	5266.2	7190.9	10004.9
**3**	97.2	491.3	1035.5	2573.0	5373.5	7368.8	9761.1
**Mean ± SD**	96.4±1.6	501.0±13.9	1047.8±16	2460.4±125.5	5295.8±63.8	7263.8±93.1	9968.4±197.7
**% CV**	1.7	2.7	1.5	5.1	1.2	1.2	1.9
**% Deviation**	3.5	-0.2	-4.7	1.5	-5.9	3.1	0.3

SD: Standard Deviation; % CV: Coefficient of Variation = (standard deviation/mean)*100; % Deviation was calculated as: (theoretical concentration minus calculated concentration/theoretical concentration)*100 [6]. Linearity of 30% HTC: Y = 0.5325X + 171.58 (r^2^ = 0.9958) and for 45% HTC: Y = 0.6488X + 88.33 (r^2^ = 0.9968).

Linearity for 10–50 μg/ml (data not shown) was determined to measure drug concentrations of samples obtained 3 hours after beginning ifosfamide infusion. For this range, only three disks were required from each sample with using the same extraction procedure.

#### Precision and accuracy

This method proved to be accurate and precise (Tables 3 and 4). For the intra-day assay, the lower limit quantification (LLQ) and the low, medium and high quality controls were analysed by quintuplicate in the same day (n = 5). The inter-day assay, the LLQ and all the quality controls were analysed in three consecutive days by quintuplicate (n = 15). All this was applied to both 30 and 45% haematocrit assays.

**Table 3:** Validation parameters of the analytical method by UPLC-MS/MS, to quantify ifosfamide in DBS at 30% HTC.

	LLQ (100 ng/mL)		
**Intra-day variability**	**Day 1**	**Day 2**	**Day 3**
Mean± SD (n = 5)	109.2±3.4	96.7± 5.3	102.7± 5.0
% CV	3.1	5.5	4.8
% Deviation	-9.2	3.2	-2.7
**Inter-day variability**			
Mean± SD (n = 15)	102.9 ± 6.8	N/R	N/R
% CV	6.6	N/R	N/R
% Deviation	-2.9	N/R	N/R
**Inter-day variability**	**QC1 (ng/mL)**	**QC2 (ng/mL)**	**QC3 (ng/mL)**
Mean± SD (n = 15)	306.0 ± 26.9	3930.9 ± 228.0	7519.8 ± 293.9
% CV	8.7	5.8	3.9
% Deviation	-2.0	1.7	6.0
**Intra-day variability**			
Mean± SD (n = 5) ^a^	269.3±8.2	3819.0± 167.4	7681.7 ± 263.6
% CV	3.0	4.3	3.4
% Deviation	10.2	4.5	3.9
Mean± SD (n = 5) ^b^	327.2±8.1	4135.3 ± 239.0	7479.6 ± 306.5
% CV	3.4	5.8	4.1
% Deviation	-9.08	-3.3	6.5
Mean± SD (n = 5) ^c^	316.35 ± 7.7	3830.8 ± 58.7	7293.8 ± 294.09
% CV	2.4	1.5	4.0
% Deviation	-5.4	4.2	8.8
**Stability assays**	**QC1**	**QC2**	**QC3**
**Room temperature (25** ^**o**^ **C)/ 3 days**			
Mean± SD (n = 5)	301.9±16.9	N/R	7789.8 ± 351.7
% CV	5.6	N/R	4.5
% Deviation	-0.6	N/R	2.6
**Short term stability (4h at 25** ^**o**^ **C)**			
Mean± SD (n = 5)	304.6 ± 8.7	N/R	7922.6 ± 357.1
% CV	2.8	N/R	4.5
% Deviation	-1.5	N/R	0.97
**Autosampler (8h)**			
Mean± SD (n = 5)	310.1 ± 16.5	N/R	7182.8 ± 161.0
% CV	5.3	N/R	2.2
% Deviation	-3.3	N/R	10.2
**Freeze-thaw (3 cycles)**			
Mean± SD (n = 5)	280.4 ± 7.9	N/R	7120.1 ± 124.5
% CV	2.8	N/R	1.7
% Deviation	6.5	N/R	11.8
**Processed sample (20 h)**			
Mean± SD (n = 5)	311.9 ± 16.3	N/R	7328.3 ± 483.5
% CV	5.2	N/R	6.6
% Deviation	3.9	N/R	8.4
**Long term stability (52 d at -80** ^**°**^ **C)**			
Mean± SD (n = 5)	293.7 ± 14.5	N/R	7764.4 ± 293.6
% CV	4.9	N/R	3.7
% Deviation	2.0	N/R	2.9

SD: Standard Deviation. CV: Coefficient of Variation. N/R: Not required according to the guidelines. Theoretical amounts for QC1, QC2 and QC3 were 300, 4000 and 8000 ng/mL, respectively.

**Table 4:** Validation parameters of the analytical method for to quantify ifosfamide in DBS at 45% HTC.

	LLQ (100 ng/mL)		
**Intra-day variability**	**Day 1**	**Day 2**	**Day 3**
Mean± SD (n = 5)	105.8±6.0	103.7± 3.1	96.43± 3.9
% CV	5.7	3.0	4.1
% Deviation	-5.8	-3.7	3.5
**Inter-day variability**			
Mean± SD (n = 15)	101.2 ± 5.7	N/R	N/R
% CV	5.6	N/R	N/R
% Deviation	-1.2	N/R	N/R
**Inter-day variability**	**QC1 (ng/mL)**	**QC2 (ng/mL)**	**QC3 (ng/mL)**
Mean± SD (n = 15)	320.9 ± 20.7	4006.5 ± 279.4	7343.2 ± 463.5
% CV	6.4	6.9	6.3
% Deviation	-6.9	-0.1	8.2
**Intra-day variability**			
Mean± SD (n = 5) ^a^	322.1±17.5	3782.5± 84.7	6981.62± 114.3
% CV	5.4	2.2	1.6
% Deviation	-6.0	5.4	12.7
Mean± SD (n = 5) ^b^	307.5±12.6	4061.8± 161.8	7534.5± 545.6
% CV	4.1	3.9	7.2
% Deviation	-2.51	-1.55	5.8
Mean± SD (n = 5) ^c^	337.1±21.6	4217.4± 381.9	7513.6± 442.3
% CV	6.4	9.0	5.8
% Deviation	-12.3	-5.4	6.0
**Room temperature stability (25** ^**o**^ **C)/ 3 days**			
Mean± SD (n = 5)	313.1± 25.4	N/R	7797.5± 179.7
% CV	8.1	N/R	2.3
% Deviation	-4.4	N/R	2.5
**Long term stability (52 d at -80** ^**°**^ **C)**			
Mean± SD (n = 5)	311.6± 15.3	N/R	7839.7±182.9
% CV	4.9	N/R	2.3
% Deviation	-3.8	N/R	2.0

The intra-day variability with quality controls was performed in three consecutive days, data for days 1 to 3 are depicted as a, b and c, respectively. SD: Standard Deviation. CV: Coefficient Variation. N/R: Not required according to the guidelines. Amount claimed for QC1, QC2 and QC3 were 300, 4000 and 8000 ng/mL, respectively.

#### Matrix effect

A subtle matrix effect was observed (10% suppression) in the high quality control, which has no effect on the assay, as revealed by analysing blank samples onto which ifosfamide/cyclophosphamide solution was added (at control points high and low concentrations) obtaining its corresponding analytic responses. This was compared to ifosfamide/cyclophosphamide solutions and its analytic responses. Normalised matrix factor (NMF) was obtained with the following formula:

$$\mathrm{NMF}=\frac{\left[\mathrm{Analyte matrix response}/\mathrm{Internal standard matrix response}\right]}{\left[\mathrm{Analyte solution response}/\mathrm{Internal standard solution response}\right]}$$

NMF with 30% haematocrit for the low control sample was 1.004±0.05 with a 4.9% CV, whereas the high control sample exhibited 0.9020±0.01 with a 1.1% CV. 45% haematocrit showed NMF of 1.020±0.03 and 0.9162±0.02 for low and high control samples and CV were 2.9 and 2.2%, respectively.

Results showed that NMF’s coefficient of variation calculated from the biological matrix was lower than 15%, which was the matrix effect criterion [6].

#### Carryover

**Fig 1:**
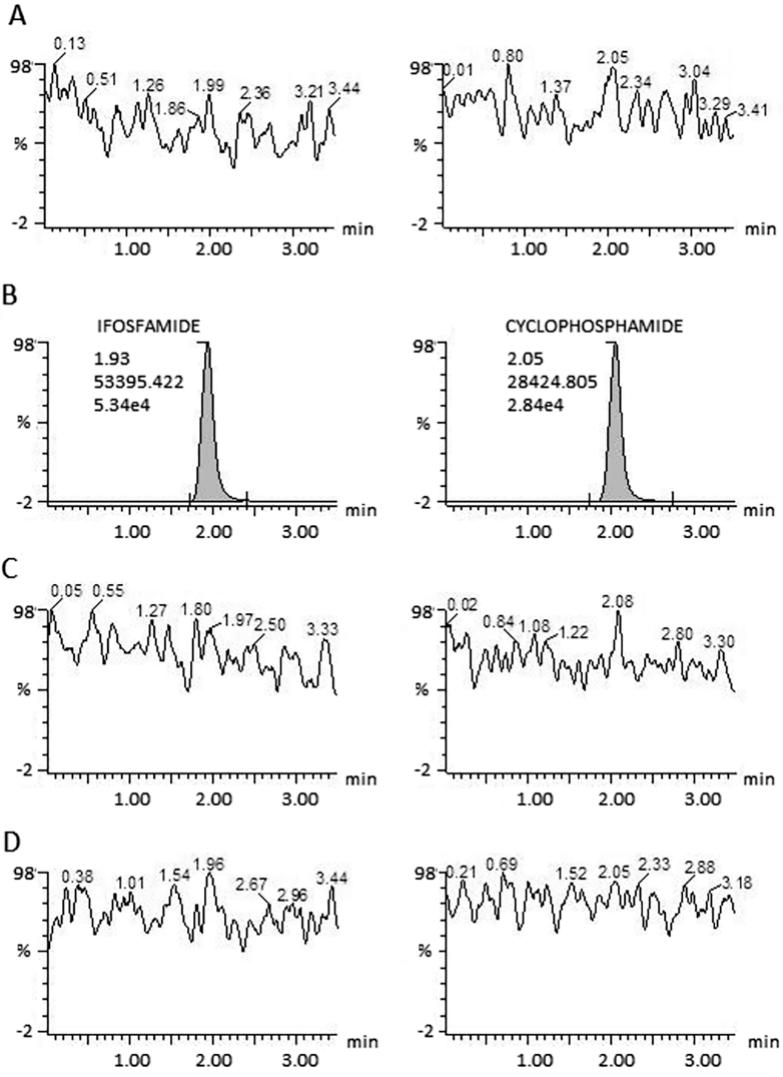
Carryover effect on ifosfamide and cyclophosphamide. Panel A shows a blank sample chromatogram previously injected. Panel B shows the higher-limit at 10000 ng/mL concentration. Panels C and D show blank samples injected after the curve higher-limit. None of the blank samples showed carryover neither for ifosfamide nor for cyclophosphamide.

There was no carryover (Fig 1) as determined by injecting three blank samples, one before and two after injecting the superior limit for quantification (10000 ng/mL). This test was done by triplicate.

#### Selectivity

**Fig 2:**
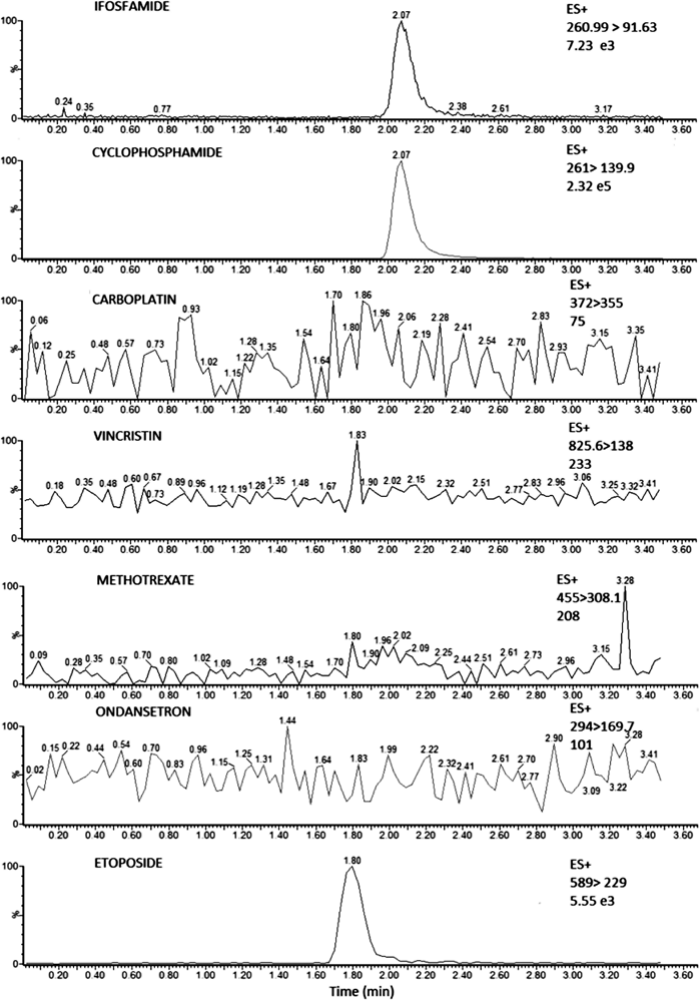
Chromatograms for multiple reaction monitoring (MRM) of individual channels. The figure shows Ifosfamide and cyclophosphamide on 24-h samples. Selectivity to other drugs administered with ifosfamide: vincristine, ondansetron, etoposide, carboplatin and methotrexate.

This method was selective to drugs administered along with ifosfamide, which were: etoposide, carboplatin, vincristine, methotrexate and ondansetron. We prepared samples with the intermediate calibrator concentration (Ifosfamide 2500 ng/mL with 30 and 45% HTC) and we added 20μL from a solution of each standard described on Table 5. The procedure was made by triplicate. Chromatograms were obtained for each drug (Fig 2) with their respective transitions (Table 5). Etoposide could be extracted with the same method, however it did not interfere with ifosfamide quantification.

**Table 5:** Selectivity: Drugs concomitantly administered with ifosfamide.

Drug administered	Drug concentration (mg/mL)	Molecular ion m/z ratio	Fragment ion m/z ratio	30% HTC Mean ± SD %CV	45% HTC Mean ± SD %CV
Etoposide	0.24	589.0	229.0	2502.3±177.9 7.1	2524.5± 87.6 3.4
Carboplatin	10.00	372.1	355.0	2513.1± 200.0 7.9	2460.8±45.1 1.8
Vincristin	0.10	825.6	138.0	2364.87±152.2 6.4	2501.8±40.1 1.6
Methotrexate	11.50	455.2	308.2	2394.9± 88.5 3.7	2551.8±150.0 5.8
Ondansetron	0.25	294.2	169.7	2594.1± 175.9 6.7	2627.7±126.0 4.8

The concentration of each drug was determined according to which it is administered in clinical practice. SD: Standard Deviation calculated from three independent repeats (n = 3).

#### Stability

Ifosfamide (1 mg/mL in MeOH) was stable in solution for at least 30 days at 4°C (data not shown) and it was also stable for the entire processing method (4 hours) and for up to three days at room temperature. It was also stable for eight hours at 15°C in an autosampler and 20 hours stored at 4°C, extracted but not resuspended. It was also stable after three freeze-thaw cycles and for up to 52 days at -80°C (Table 3).

### Method application

**Fig 3:**
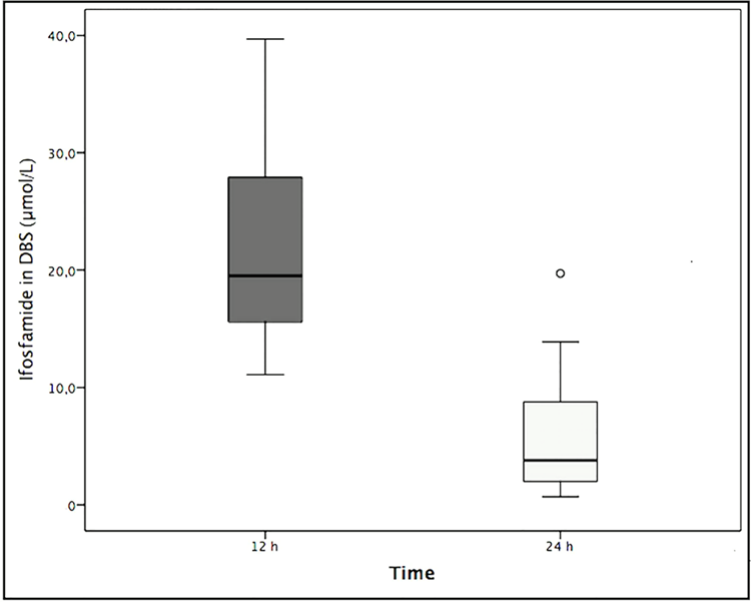
Comparison between medians for blood ifosfamide levels obtained from DBS. The figure shows a comparison between medians for blood ifosfamide levels obtained from DBS after 12 hours (dark gray box) and 24 hours (Light gray box), p <0.0001.

Monitoring of ifosfamide blood concentration was made in 14 paediatric patients (9 males and 5 females) with solid embryonic tumours. The median for ifosfamide concentration in dried blood spots at 12 hours was 19.5 μmol/L (Q_25_ 14.4–Q_75_ 29.0) and was compared to that at 24 hours, which was 3.8 μmol/L (Q_25_ 1.5–Q_75_ 9.9) showing a statistically significant difference (p = <0.0001 (Fig 3). Concentrations ranged from 11.1–39.7 μmol/L in 12-hour samples whereas 24-hour samples did from 0.7–19.7 μmol/L (Table 6).

**Table 6:** Demographic data and ifosfamide levels from patients, measured on DBS after 12 and 24 hours.

Patient	Age(Y)	Gender	Weight (kg)	Diagnosis	Course	12 h sample		24 h sample	
						ng/mL	μmol/L	ng/L	μmol/L
1	5	M	18.0	Retinoblastoma	6	3380.4	12.9	244.3	0.94
2	17	M	55.0	Germ cell tumour	1	2903.8	11.1	34.8	0.13*
3	3	M	13.0	Sarcoma	4	5813.5	22.2	212.7	0.81
4	14	M	49.0	Sarcoma	2	16558.0*	63.4	3644.3	13.95
5	15	F	44.6	Astrocytoma	1	9218.0	35.3	999.1	3.82
6	12	F	55.0	Astrocytoma	1	6706.1	25.6	2915.5	11.16
7	15	M	93.0	Sarcoma	3	7870.4	30.1	5157.5	19.75
8	11	F	51.7	Ewing Sarcoma	2	5103.1	19.5	773.1	2.96
9	3	F	13.3	Sarcoma	3	4134.5	15.8	519.4	1.99
10	0.9	M	8.0	Retinoblastoma	1	4075.8	15.6	1150.4	4.41
11	2	F	12.6	Germ cell tumour	1	4084.6	15.6	187.4	0.72
12	0.3	M	4.7	Sarcoma	1	10377.9	39.7	2291.6	8.77
13	16	M	54.7	Osteosarcoma	1	7288.3	27.9	1723.1	6.59
14	9	M	32.3	Hepatoblastoma	4	3477.5	13.3	689.8	2.64

M: Male, F: Female, Y: years.

* Value out of the range. It was estimated by extrapolating it from the curve and it was not included for calculating the median value.

## Discussion

A number of analytical methods and detection techniques have been developed to measure blood levels of ifosfamide, most common one being liquid-to-liquid [9–11], though derivatisation [12], protein precipitation [13] and solid phase extraction [14–16]. Detection has been very diverse and has included gas chromatography [17], UV detection with liquid chromatography [11, 12, 18] and mass spectrometry detection (tandem, time of flight, gasses) [9, 10, 12, 19, 20]. In the present study, we describe the development and validation of a new method to quantify ifosfamide using dried blood spots in Guthrie cards, originally developed to screen newborn diseases, which are very smart for children and show a great advantage over other quantification methods that require at least 100 μL or even 1 mL blood. Another noted advantage is the minimum amount of solvents used and the short time needed for the analysis, which translates into a significantly lower cost.

DBS technique is better accepted among the patients, both children and their responsible relatives, in comparison to other procedures such as venepuncture which sometimes requires intravenous catheterization with anticoagulant measures and compromises the patient’s mobility. Although it is true that a finger prick can cause mild discomfort, it is much less invasive and less painful than direct venepuncture, with the additional advantage of a lesser or null risk of inducing infections. That is why we consider this method to be better when used in paediatric clinical practice, especially in those undergoing antineoplastic therapy.

Furthermore, our method has been validated for monitoring blood ifosfamide concentrations in paediatric patients with solid embryonic tumours, which is clinically relevant because it can help determining optimal ifosfamide therapeutic concentrations and the threshold for dose-limiting side effects and toxicity, which still remain unknown in paediatric patients.

Since our method has been suitable to paediatric patients, it could be implemented to estimate the therapeutic levels of ifosfamide just as it is commonly done with other drugs, such as methotrexate. To our knowledge, children treated with methotrexate are in high risk of developing adverse effects such as mucositis or neutropenia if methotrexate plasma levels are higher than 1 μmol/L, 42 hours after the time of infusion [2]. Such criterion has allowed physicians to promptly provide rescue measures (such as folinic acid administration) if toxic levels are reached, avoiding neutropenia, mucositis, and other side effects.

Ifosfamide blood levels have shown a significant interindividual variability, both in adults and paediatric patients [21–23]. Likewise, ifosfamide blood levels observed in the present study showed great interindividual variability which could play a key role in clinical response to treatment.

Interindividual variability could be influenced by several factors that affect ifosfamide metabolism such as: self-induction, drug interactions and/or polymorphisms in genes that code for enzymes that transport or metabolise ifosfamide [24]. It has been reported that polymorphisms in *CYP450* coding genes, such as *CYP2B6* could determine variations in the expression and activity of the enzyme and therefore contribute to the variability of ifosfamide levels between patients with clinically relevant differences [25]. In addition, with this developed and validated method it could be possible to achieve an optimal pharmacologic effect with the least toxicity in the least amount of time, which could accelerate patient’s recovery and reduce in-hospital mean time. This UPLC-MS/MS analytical method is linear, reproducible, exact and selective, and it showed to be fast and feasible with a blood sample collection minimally invasive. This method is applicable to determine ifosfamide blood levels routinely, for children with solid malignancies undergoing treatment with ifosfamide.
